# Effects of stimulus and task structure on temporal perceptual learning

**DOI:** 10.1038/s41598-020-80192-6

**Published:** 2021-01-12

**Authors:** Rannie Xu, Russell M. Church, Yuka Sasaki, Takeo Watanabe

**Affiliations:** Department of Cognitive, Linguistic and Psychological Sciences, Brown University, Providence, 02912 USA

**Keywords:** Human behaviour, Learning and memory

## Abstract

Our ability to discriminate temporal intervals can be improved with practice. This learning is generally thought to reflect an enhancement in the representation of a trained interval, which leads to interval-specific improvements in temporal discrimination. In the present study, we asked whether temporal learning is further constrained by context-specific factors dictated through the trained stimulus and task structure. Two groups of participants were trained using a single-interval auditory discrimination task over 5 days. Training intervals were either one of eight predetermined values (FI group), or random from trial to trial (RI group). Before and after the training period, we measured discrimination performance using an untrained two-interval temporal comparison task. Our results revealed a selective improvement in the FI group, but not the RI group. However, this learning did not generalize between the trained and untrained tasks. These results highlight the sensitivity of TPL to stimulus and task structure, suggesting that mechanisms of temporal learning rely on processes beyond changes in interval representation.

## Introduction

Temporal perceptual learning (TPL) refers to a training-related improvement in the detectability and discriminability of temporal intervals^1^. This type of learning can be long-lasting^2^ and is generally thought to reflect a refinement in the representation of a learned interval^3–6^. Typically, TPL do not generalize to untrained conditions which differ from the trained temporal interval^3–8^. For example, Wright et al.^9^ found that auditory discrimination training using a 100 ms tone selectively improved the discriminability of the trained interval, but this improvement did not generalize towards any neighboring conditions (e.g., 50 ms, 200 ms, 500 ms). Observation of temporally-specific learning is of key importance from a theoretical standpoint because it suggests that the mechanisms of TPL might involve duration-selective tuning mechanisms in sensory and/or association cortices of the brain^10–12^.

In addition to training-related refinements in temporal representation, TPL can similarly affect the way we respond to a temporal stimulus through non-temporal factors. For instance, optimizations in task-related processing (e.g., working memory capacity or decision bias) could change how sensory information is being interpreted in forming a behavioral choice; consequently, perceptual learning would rely on a strengthening in the relationship between an external stimulus and the optimal decision in a perceptual task^13–15^. In this view, TPL cannot exclusively reflect changes in the representation of a temporal interval, but also in the way we make decisions about that interval within a specific temporal task. Learning must therefore be *task-specific* and confined within the trained structure of information^16^—featural learning by itself is insufficient to account for the behavioral improvements observed with practice^17–19^.

Compared with perceptual learning in the visual modality (VPL), evidence of task-specificity in the temporal domain has been limited to a small number of studies exploring cross-modal effects of learning, and often producing mixed results^4,20–23^. Even within single studies, evidence of bi-directional transfer between the auditory and visual modalities have been inconclusive^24,25^. One interesting exception to this is a study by Meegan et al.^20^ where training on a temporal discrimination task was found to improve motor production of the same interval. However, this type of task-independent transfer has not been documented within the same task type (e.g., strictly sensory or motor), and might be asymmetrical in nature. Therefore, it is unclear whether TPL is also accompanied by changes in task-specific processes.

The purpose of the present study is to abridge findings between VPL and TPL through a systematic investigation of task-specificity in temporal learning. If we assume that TPL shares a similar mechanism of learning to VPL in that performance improvement in perceptual tasks reflect changes to (1) a learned stimulus/feature and (2) the way we respond to the stimulus/feature within a task, we would expect TPL to be specific to the response strategies and demands within a learned task (i.e., no learning transfer to an unlearned task). To this end, we employed a similar pretest-training-posttest design as Meegan et al.^20^, where training involves extensive practice on a temporal discrimination task, and a pretest/posttest session which examines transfer of learning to an untrained task. Since we used the same temporal interval in both tasks, our design allows for an independent treatment of *interval-specific* versus *task-specific* learning. Further building off the results of Meegan et al.^20^, the use of two perceptual discrimination tasks in the present study is a more sensitive indicator of task-specificity in TPL, as it does not involve confounding factors such as motor efficiency or modality dominance in temporal processing^24^.

A second way in which we sought to investigate task-specificity in TPL concerns regularity in the stimulus distribution during training. One of the recent findings in VPL suggests that the degree of stimulus uncertainty or the ability to discern statistical regularities in the training stimulus might influence the type of decision strategy that is adopted by the viewer^26–28^. Consistent with this idea, we predict that TPL should be similarly sensitive to the stimulus structure during training since perceptual learning reflects a highly context-specific experience. As such, we also varied the type of stimulus input used during training. Half of the participants in our experiment received 8 fixed intervals (FI group) while the other half received random intervals (RI group) which varied from trial to trial with minimal certainty. We predict that the group receiving fixed input during training will show an improvement in discrimination performance, while the group trained using random intervals—as a result of lower statistical regularities in the training input—may not be able to adopt a response strategy appropriate to the task. In summary, we expect improvements in temporal discrimination to be strongly coupled to the trained structure of information, with no generalization beyond unlearned stimulus and task dimensions, akin to what is commonly demonstrated in VPL.

## Results

### Learning in the trained task

**Figure 1:**
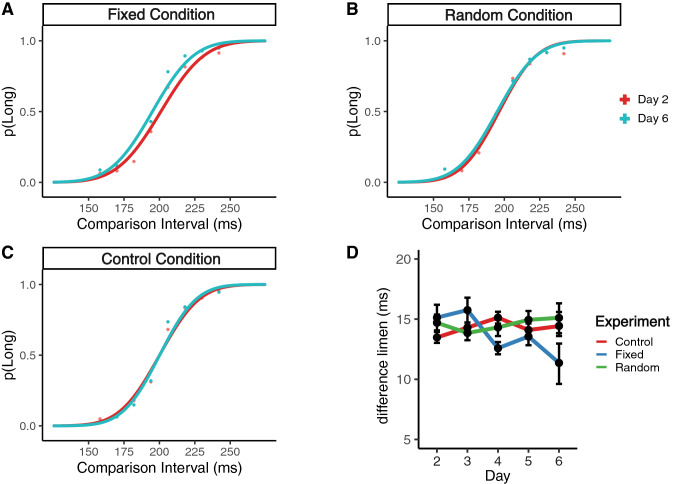
(**a**–**c**) Changes in the psychometric function in each group before and after training. (**d**) Changes in the difference limen (ms) across days. Error bars represent ± SEM.

To assess the amount of training-related change in performance between the FI and RI groups, we fit psychometric functions using the proportion of “long” responses at each comparison interval using the Quickpsy package^29^ in R (Fig. 1). Based on the goodness-of-fit indicated by the coefficient of determination (r^2^), we excluded data from three outlying subjects.

**Figure 2:**
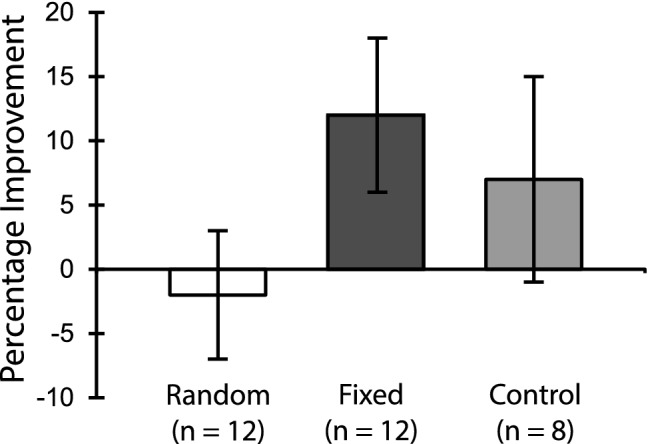
Percentage improvement on the trained temporal discrimination task. Percentage improvement is calculated from the change in difference limen between the first and last day of training (day 2–day 6), divided by an individual’s performance on the first day of training (day 2): %Improvement = (day 2 − day 6)/day 2 × 100. This calculation measures the amount of improvement from baseline relative to an individual’s initial performance on the task. Error bars indicate between-subjects ± SEM.

To investigate the effects of varying stimulus structure on TPL, we compared changes in timing ability of the RI and FI groups between the first and last day of training. We submitted the DL values to a 2 × 2 mixed ANOVA with group (FI/RI) as a between-subjects factor and session (day 2/day 6) as the within-subject factor. Overall, DL values decreased with training (F_1,22_ = 4.83, *p* = 0.038, η^2^ = 0.09; Day 2: M ± SEM = 15.2 ± 10.2; Day 6: M ± SEM = 12.9 ± 6.6), with no significant differences between RI and FI (F_1,22_ = 1.96, *p* = 0.17). However, we did observe a significant interaction effect between group and session (F_1,22_ = 4.41, *p* = 0.047, η^2^ = 0.08). A post-hoc pairwise t-test on the percentage change in DL between the FI group and the RI group revealed a significant selective improvement with training in the FI group (t_11_ = 2.17, *p* = 0.041), but not the RI group (t_11_ = 0.16, *p* = 0.87). To further qualify the degree of change in DLs within each group, we calculated a percent change in individual timing performance based on their initial performance on the first day of training (Fig. 2). For the FI group, which showed an average of 12% decrease in the DL across subjects, there was a significant learning effect as compared with the RI group with only an average of -2% change. These results are consistent with the idea that practice improves auditory bisection performance when a group receives a preset number of training intervals, but not when the test intervals belong to a random distribution. Our results suggest that TPL is similar to VPL in that learning is dependent on the input structure of information during training, and that the degree of improvement might be influenced by our ability to discern statistical regularities in the training stimulus^30^.

To further understand how statistical regularity contributed to the selective performance improvement in the FI group, we recruited nine additional participants for a control condition. In the RI group, every test interval was selected at random from a normal/gaussian distribution with a mean of 200 ms. This entails that a greater number of values are closer to 200 ms than any other value within that range. In contrast, the FI group always compared the reference against 1 of 8 fixed intervals with equal (uniform) probability. The use of a fixed number of values therefore ensures a greater degree of statistical regularity both through (1) the stimulus distribution, as well as (2) an overall smaller number of possible interval values—either/both of which might have contributed to the observed differences in learning between groups. If the latter is true, changing the stimulus distribution of the RI group from gaussian to uniform would have minimal effect on learning since the overall degree of certainty/variability of the training stimulus remains much lower than the FI group.

### Control group

To address this possibility, we recruited a control group whereby all stimuli and parameters are identical to the RI condition, with the exception that test intervals are now drawn from a uniform—not gaussian—distribution. If TPL relies on regularities in the training stimuli, our manipulation would serve to equate the frequency of occurrence for all target intervals, leading to a similar behavioral improvement as observed in the FI group.

Once again, we fit individual psychometric functions for each session, and calculated a percentage change in DL. In the control group, we did not find a significant change in performance on the first and last day of training (t_7_ = 1.22, *p* = 0.26), replicating results of the RI group. To see if there were any differences in learning between all three groups, we conducted a separate mixed model ANOVA with group (FI/RI/Control) and session (day 2/day 6) as factors. In this analysis, there was once again a significant, though reduced effect of training (F_1,29_ = 4.21, *p* = 0.049, η^2^ = 0.06), which likely reflects the lack of change in DL values in the RI and control groups. Also consistent with our prior results, we observed a significant group by session interaction (F_2,29_ = 3.57, *p* = 0.041, η^2^ = 0.09), and no differences between groups (F_2,29_ = 0.12, *p* = 0.88). These results again argue against the hypothesis that learning in the FI group is attributable to differences in the stimulus distribution (uniform vs. gaussian) during the training phase.

### Generalization to the untrained task

**Figure 3:**
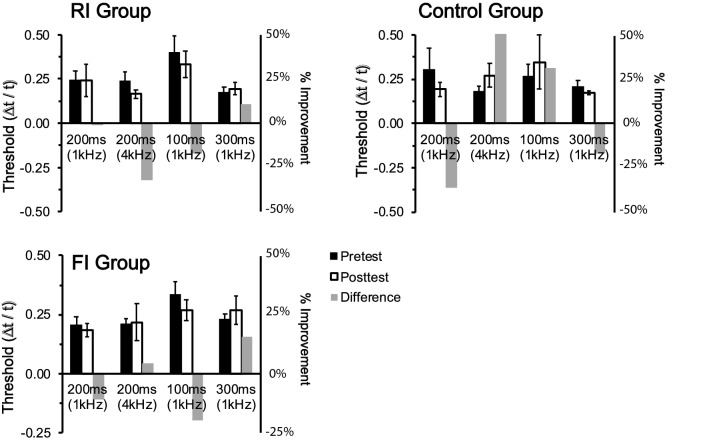
Discrimination thresholds (left) and percent improvement (right) for the untrained temporal comparison task. We did not observe a significant learning effect for the trained 200 ms interval between pretest and posttest sessions. Error bars represent ± SEM.

In addition to learning in the trained task, we also tested generalization of learning using an untrained discrimination task with the same reference interval. To compare discrimination performance across the 4 interval conditions (100 ms, 200 ms, 300 ms at 1 kHz and 200 ms at 4 kHz), Weber’s Fractions (WF) were used (Fig. 3).

We then submitted the WFs to a 3 × 2 × 4 mixed model ANOVA with group as a between-subjects factor, and session and condition as within-subject factors. Our analysis revealed a significant main effect for condition (F_3,87_ = 4.01, *p* = 0.038, η^2^ = 0.05), with the 100 ms condition having significantly higher discrimination thresholds during both pretest (WF_pretest_ ± SEM = 1.29 ± 0.011) and posttest (WF_posttest_ ± SEM = 1.42 ± 0.026) sessions than all other interval conditions—a finding commonly reported in other TPL studies^23,24,31^. Importantly, we did not observe a significant effect of training (F_6,87_ = 0.88, *p* = 0.45), nor group by condition by session interaction (F_6,87_ = 1.39, *p* = 0.22). Together, these results suggest that given our current paradigm, training-related improvements on a temporal task does not generalize to an untrained task even if the interval used between the two tasks are identical. Of course, we must recognize that the two paradigms used in our study (i.e., temporal bisection and discrimination task) may not fully capture learning transfer across all types of temporal tasks, and that various task-related differences in structure, procedure, and design could potentially contribute to the null transfer observed in our experiment. However, our results provide an initial demonstration of task-specificity in TPL, possibly representing an additional constraint in the mechanisms of temporal learning.

## Discussion

Performance on a variety of perceptual and motor timing tasks can be improved with training^3,9,20,32^. This learning is largely interval-specific^3–8^, which motivates the view that TPL enhances the featural representation of a temporal interval. In contrast, relatively little is known about the degree to which non-temporal components of a task affect TPL. The present study explores the influence of stimulus- and task-specificity on temporal learning. In our experiment, we demonstrate that: (1) learning is sensitive to statistical regularities in the training stimulus—only the FI group, trained using a fixed number of interval values, showed a significant improvement in temporal discrimination; and (2) learning is specific to the response strategies of a trained task, with no generalization of learning to an untrained task.

We speculate that there are a few factors contributing to the selective improvement for the FI group, but not the RI group. Since the to-be-learned interval (i.e., 200 ms) was identical in both groups, this difference in learning can first and foremost be explained through differences in response strategy. For example, in lieu of comparing each test interval against the reference interval, participants in the FI group might have opted to learn a specific set of decision weights based on the optimal stimulus–response relationship. In other words, TPL reflects the learning of the most appropriate response given each of the 8 fixed values. With a finite number of possible values, the FI group can effectively adopt this strategy. By comparison, the RI (and control) group was not able to extract any meaningful relationships based on an infinite number of possible intervals and their associated responses. This suggests that mechanisms of temporal learning might involve an optimization of connections between stable stimulus representations and relevant task decisions.

A related consequence of being exposed to greater variability in training input is the possibility of resulting differences in memory quality for the reference duration. Since the RI group was exposed to a greater number of test intervals during training, it is likely that their representation of the 200 ms reference is inherently noisier and more easily contaminated by additional input. As a result, degradations in memory may have contributed to the observed differences in learning across groups. This interpretation is again consistent with the stipulation that top-down processes are necessary in perceptual learning because it suggests that higher cognitive functions, such as attention and memory factors may influence perceptual processing at a very early stage of learning. With both considerations, our results suggest that TPL cannot rely *exclusively* on changes at the level of stimulus/interval representation, otherwise our manipulation of the comparison interval would not play a role in learning. Instead, we speculate that higher-level processes must also be involved in the learning process, and it is this set of task-specific strategies that also become improved with training.

More broadly, our finding that TPL is sensitive to regularities in the training stimulus is consistent with what is often reported as “roving effects” in VPL^28^. With roving, the use of interleaved visual stimulus or task structures can impair learning in a number of visual discrimination tasks^26–28,33^. This interference is suggested to result from activity in overlapping neural populations, and arising only when there is a high degree of similarity between a trained stimulus type and task^34,35^. In our study, the observation that learning was impaired for the RI group receiving interleaved training intervals therefore highlights a crucial similarity in the underlying learning mechanisms between temporal and visual perceptual learning. A recent finding by Parkosadze et al.^36^ showed that perceptual training under roving conditions may be mitigated by a more extensive training regimen. Similarly, we might expect to see comparable learning outcomes for the RI group with a greater number of training trials.

Another novel finding in our study is the specificity of learning within a trained temporal task. For the FI group, improvements in temporal discrimination in the practiced task was not transferred to the unlearned comparison task, even with the same reference interval. While our results differ from learning generalization reported in previous studies, one way to reconcile these discrepant findings is by considering the ease of generalization between trained and untrained conditions. Specifically, we speculate that learning generalization may be inversely proportional to task difficulty during training. In Buonomano et al.^37^ for example, training using a short interstimulus interval (ISI) resulted in learning specificity for the trained condition, whereas the use of a longer ISI was able to benefit temporal discrimination in untrained conditions^37^. Similarly in Chen and Zhou^38^, transfer effects were robust for a visual Ternus display when trained using auditory/tactile intervals, but similar benefits were not observed in the visual modality alone. These results are consistent with the idea that learning generalization increases with training^5^, and that practice in a more difficult or cognitively demanding condition would impair transfer to untrained conditions. Conversely when training is conducted using easier (e.g., longer ISI) or modality-dominant (e.g., auditory) stimuli, learning transfer is typically observed. This suggests that differences in the relative ease in accessing unisensory temporal information can predict intermodal generalization patterns in untrained conditions.

Despite these considerations, we must acknowledge the possibility that the learning specificity reported in the present study could be attributable, at least in part, to differences in methodology used to estimate the discrimination threshold between the trained and untrained tasks. Since the testing phase involves an adaptive procedure (AP) rather than the method of constant stimuli (MCS), it is possible that our null transfer results reflect an insensitivity of one estimation method over the other, rather than an inability to generalize learning across tasks. For instance, since the AP involves the comparison of two interval durations, between which the difference is changed adaptively on every trial, this ever-changing difference could evoke the use of subtle irrelevant cues in interval discrimination^39^ that is not available in MCS procedures. While both the AP and MCS are commonly used to estimate temporal discrimination performance in TPL research^1^, a direct comparison between the two methods have not been attempted, so we must recognize that inherent differences in threshold estimation may contribute to the learning specificity observed here.

In summary, our finding of task-based specificity is best understood as an additional constraint on the mechanisms of TPL. Based on several models in VPL, specificity in learning could reflect changes in a trained feature (i.e., reference interval) given a specific set of task- and context-specific demands^40–43^. Consistent with this hypothesis, our results extend our current understanding for the mechanisms of TPL by constraining interval-specific learning within a broader framework of task-related processes.

## Methods

### Participants

Twenty-nine right-handed adults (16 females; mean age: 23.8 ± 3.9 years) with normal or corrected-to-normal vision and hearing were recruited for participation in our experiment. We justified our sample size using the G*Power software^44^ with a medium effect size of 0.40 and a power estimate of 0.80. A total of 24 subjects were determined to be necessary for significance in a mixed-model ANOVA with this analysis (2 groups, F_critical_ = 1.05, non-centrality parameter = 184.32). Two participants were unable to complete the experiment due to scheduling conflicts, and 4 more outliers (3 from the FI/RI group, 1 from the control group) were excluded from analysis due to abnormally high thresholds during training (> 2.5SD from the group mean). For the control experiment, nine new participants were recruited from the same subject population (8 females; mean age: 22.4 ± 2.9 years). Each session was held at the same time each day to avoid possible confounding effects of time of day on temporal processing^45^. Informed consent was obtained in writing from each subject prior to commencing the study and approved by the institutional review board (IRB) at Brown University.

### Stimulus and apparatus

Participants were seated in a sound-insulated room with dim lighting. All stimuli were generated and presented using MATLAB with Psychophysics Toolbox extensions, version 3.0.14^46^ and presented on a ViewSonic—VA2226w monitor, measuring 20 × 14 in., with a refresh rate of 75 Hz and a viewing distance of approximately 38 cm. Auditory stimuli were presented at 86 dB SPL through noise-cancelling Sennheisser headphones and included a 5 ms on and off ramp. All responses were collected using a standard US keyboard.

### Procedure

**Figure 4:**
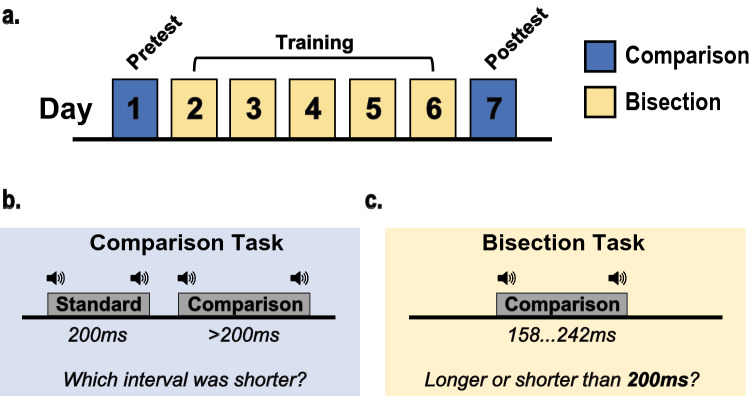
(**a**) Experimental and task procedure. (**b**) Design for the untrained comparison task. Participants indicated which of two auditory tones was longer in duration (i.e., the comparison interval). Presentation order was randomized on every trial. (**c**) Design for the trained bisection task. Participants indicated whether the comparison interval was longer or shorter than 200 ms.

We used a standard pretest-training-posttest design over seven consecutive days (Fig. 4a). During the training phase (Days 2–6), each participant completed 2400 trials of a single-interval temporal discrimination task. We justified the number of practice trials under the guidance of previous studies which have demonstrated substantial learning effects using ~ 2500 trials^6,20,24^, and with as little as a single day of training with 900 trials^32^. At the beginning of each session, participants were presented with a 200 ms tone at 1 kHz and instructed to categorize subsequent intervals as being either “longer” or “shorter” than the reference (Fig. 4c). In the training phase, response feedback was provided immediately after every response by the participant. For the RI group, comparison intervals were drawn from a gaussian distribution with a mean of 200 ms and minimum and maximum bounds of 158 ms and 242 ms, respectively. In the FI group, 1 out of 8 predetermined interval values (158, 170, 182, 194, 206, 218, 230, 242 ms) were selected at random, with equal probability. Similar to the RI group, the mean and range of these intervals were 200 ms and 158–242 ms, respectively.

During the testing phase (Days 1 and 7), discrimination thresholds were measured using an adaptive staircase procedure^47^ with four conditions: 100 ms, 200 ms, 300 ms at 1 kHz, and 200 ms at 4 kHz. The presentation order for each condition was pseudo-randomized according to the Latin Square design for each participant and kept constant between pretest and posttest sessions. The task involves a two-interval temporal comparison task (Fig. 4b) with a 3-down, 1-up adaptive staircase. Each training session consisted of 80 trials per test condition, and similar to estimation methods of previous studies^9,48^, we calculated discrimination thresholds based on the average of last 6 reversals within each block. On each trial, two auditory intervals were presented: a standard (t) and a comparison (t + Δt). Listeners must indicate which interval was the standard (i.e., shorter interval) by pressing either the “J” or “K” keys using their index and middle finger. The length of the standard and comparison was identical on the first trial of every block, forcing subjects to guess. The step size for the first five reversals is 5% of the standard and 1% thereafter. The inter-stimulus interval was jittered to minimize predictability between the offset of the first interval and the onset for the second interval. All methods and procedures were performed in accordance with the relevant guidelines and regulations set out by the IRB and human research protection program (HRPP) at Brown University.

### Data analysis

For the training phase, the comparison intervals for the random condition were first divided into 8 equal bins such that the mean value for each bin corresponds to the comparison values in the fixed condition. It should be noted that the number of trials in each bin is different given the Gaussian distribution used in the RI condition. This discrepancy is addressed in our control group, which employs a uniform distribution similar to the fixed group (see “Results” section).

For each bin, we first calculated the proportion of “long” responses by dividing the number of times the participants responded “longer” to a given comparison value by the total number of responses within that bin (i.e., number of times the participant replied “short” plus number of times the participant replied “long”). Next, we fit a psychometric function using the quickpsy package in R, allowing for lapses^49^. This function showed a monotonic increase with duration as participants generally responded “short” to the shortest duration and “long” to the longest durations. Based on these fitted functions, we then computed the just-noticeable-difference or the difference limen (DL) using the formula:

$$DL=\frac{{x}_{75\%}- {x}_{25\%}}{2}$$

where *x* represents the length of the comparison interval that yielded 25% and 75% “longer” responses, respectively. The DL therefore represents the steepness of the slope on the psychometric function and estimates the differential sensitivity of an observer during a timing task^50^. In this calculation, a smaller DL corresponds to a steeper psychometric function and a greater ability to discriminate between more similar intervals.

For the task used in the pretest/posttest sessions, we used a standard estimation technique for the discrimination threshold based on the average of the last 6 reversals in the staircase procedure. Since there were 4 different interval conditions, we then normalized each threshold measure by the reference duration. This is reported as the Weber’s Fraction (WF), with a smaller value corresponding to a higher degree of discriminability^51^:

$$\mathrm{WF}=\frac{\Delta t}{reference\,duration}$$

where Δt represents the average difference between the comparison and reference intervals (in ms) on the last 6 reversals of the 1-up, 3-down staircase procedure. For instance, if the value of Δt is 40 ms for the 200 ms condition, it suggests that they are able to discriminate between 200 ms (reference) and 240 ms (comparison) with ~ 79% accuracy. We can then compute their WF by dividing the threshold by the interval condition (200 ms), resulting in a WF of 0.2. The use of the WF has the added advantage of comparing timing sensitivities across multiple conditions with different base reference values.
